# Danger Comes from All Fronts: Predator-Dependent Escape Tactics of Túngara Frogs

**DOI:** 10.1371/journal.pone.0120546

**Published:** 2015-04-15

**Authors:** Matthew W. Bulbert, Rachel A. Page, Ximena E. Bernal

**Affiliations:** 1 Behavioural Ecology Group, Department of Biology, Macquarie University, North Ryde, New South Wales, 2109, Australia; 2 Smithsonian Tropical Research Institute, Apartado 0843–03092, Balboa, Ancón, Panamá, República de Panamáa; 3 Department of Biological Sciences, Purdue University, West Lafayette, Indiana, 47907–2054, United States of America; Brown University, UNITED STATES

## Abstract

The escape response of an organism is generally its last line of defense against a predator. Because the effectiveness of an escape varies with the approach behaviour of the predator, it should be advantageous for prey to alter their escape trajectories depending on the mode of predator attack. To test this hypothesis we examined the escape responses of a single prey species, the ground-dwelling túngara frog (*Engystomops pustulosus*), to disparate predators approaching from different spatial planes: a terrestrial predator (snake) and an aerial predator (bat). Túngara frogs showed consistently distinct escape responses when attacked by terrestrial versus aerial predators. The frogs fled away from the snake models (Median: 131°). In stark contrast, the frogs moved toward the bat models (Median: 27°); effectively undercutting the bat’s flight path. Our results reveal that prey escape trajectories reflect the specificity of their predators’ attacks. This study emphasizes the flexibility of strategies performed by prey to outcompete predators with diverse modes of attack.

## Introduction

Prey use a diverse range of anti-predatory strategies [1]; foremost among them is minimising predator detection [1–3]. Once prey are detected by a predator, however, fleeing is generally the prey’s last recourse [1,4]. The success of this flight behaviour is ultimately determined by when [1–8] and where [1,9–12] prey choose to flee. The factors that determine when prey are likely to flee have been well studied across a wide variety of taxonomic groups. In contrast, the importance of the direction or rather the escape trajectory used by prey has only more recently been recognised [1,10,12,13].

The importance of selecting the ‘best’ escape trajectory is no more apparent than when prey cannot outpace their attacker [14, 15]. Barn owls (*Tyto alba*), for instance, move swiftly and quietly towards spiny mice (*Acomys cahirinus*). Spiny mice do not have the straight-line speed to outrun a swooping barn owl [14]. Instead the mice typically flee at last moment using an escape angle perpendicular to the flight path of the owl. Moving sooner and/or choosing an angle either, away or, towards the owl have been shown to substantially increase the likelihood of prey capture. [15]. This owl-mouse example is just one of several examples of where, using a particular escape trajectory, is predicted to maximise escape success from a single predator [12,16]. Prey, however, are typically confronted with multiple predators, which often use divergent hunting behaviours and strategies. Accordingly, prey should use escape tactics that best match their predators’ strategies if they are to maximise their chances of survival [17]. Prey exposed to predators with different attack methods, therefore, are expected to display equally diverse predator-specific escape behaviours.

It is well recognised that some prey vary their responses to different predators using behaviours ranging from freezing to fleeing [13,14]. Woodmice (*Apodemous mystacinus*), for example, run when exposed to most predators [18,19] but either freeze or jump in the presence of stoats [20]. However, the more subtle multi-predator escape strategy, of a prey varying its escape trajectories in response to different predators, has yet to be described. Nonetheless different escape trajectories are predicted when prey is attacked by predators that approach from vastly different spatial planes, as in the case of aerial versus terrestrial predators. Both aerial and ground-dwelling prey evade aerial attackers by often fleeing perpendicular to the attack trajectory (e.g. Spiny mouse *Acomys cahirinus* [14]; Blue tit *Parus caeruleus* [21]). In contrast, prey species attacked by ground-based predators generally move away from their assailants (e.g. reviewed in [12] and references therein). To our knowledge, however, no study has empirically investigated the escape trajectory of the same prey species to predators with different attack strategies. Here we examine the flexibility in escape trajectories of a ground-dwelling prey species, the túngara frog (*Engystomops pustulosus* (Shreve 1941), formerly *Physalaemus pustulosus*), to attacks by predators with distinct hunting behaviours (aerial and terrestrial).

Túngara frogs are small palatable anurans that are abundant and widespread in Middle America. Male túngara frogs aggregate in temporary ponds to advertise for females, but their calls make them vulnerable to being preyed on by a variety of predators. Fringe-lipped bats, *Trachops cirrhosus*, attack túngara frogs from the air using the advertisement calls of male frogs to detect and localise them [22,23]. Túngara frogs are also prey for numerous ground-based predators including cat-eyed snakes (*Leptodeira annulata*) [24], opossums (*Philander opossum*) [25]; South American bullfrogs (*Leptodactylus savagei*) [26], cane toads (*Rhinella marina*, formerly *Bufo marinus*) [27] and invertebrates such as large crabs (*Potamocarcinus richmondi*; [26] and spiders (*Sericopelma rubronitens*) [28]. The breadth of predator types that consume túngara frogs make these frogs ideal to test the hypothesis that prey use predator-specific escape responses when faced with predators with different attack trajectories. To test this hypothesis we presented calling male túngara frogs in the field to either a ground (snake model) or an aerial (bat model) predator. We predicted that túngara frogs would flee perpendicular to the approach angle of an aerial threat and escape directionally opposite from approaching terrestrial predators.

## Materials and Methods

We conducted this study in the field, around the facilities of the Smithsonian Tropical Research Institute in Gamboa, Panama (9°07.0’N, 79°41.9’W) from July to August 2011. To simulate the natural conditions under which signalling frogs are attacked, we tested túngara frogs only when calling. We found calling males in shallow puddles within drainage lines in Gamboa between 1900–2300hrs. Leaving the frogs *in-situ*, we stimulated continued calling by broadcasting a synthetic male túngara frog call played in a continuous loop. Calls were synthesised using software developed by J. Schwartz (Pace University at Pleasantville, NY, USA; sample rate 20 kHz and 8 bit). The playback stimulus consisted of bouts of the synthetic call and periods of silence such as those found in natural calling bouts of túngara frogs [29]. We used this playback stimulus only to stimulate males to call and then stopped it to allow natural calling behaviour. Once the focus male was calling consistently, we introduced a simulated predator attack. We video recorded all trials at 30 frames s^-1^ using a Sony Nightshot DCR-SR45 camcorder with Sony HVL-IRM infrared lights, and later digitised them using ImageJ v1.4u [30] for detailed analysis. We converted the videos’ time signature to seconds to determine the time between predator detection by the frog and when the frog fled. We defined this period as the time from when the frog ceased calling, after the model was introduced, to when the frog fled.

To prevent retesting, after each trial we captured and temporarily removed the tested individuals from the population. We housed captive frogs in the laboratory in a individual large plastic container (1.5x0.5x0.5m) lined with leaf litter and given water *ad libitum*. Frogs were fed every 2–3 days with live termites. When the testing of all individuals was completed, we released all frogs at their respective capture sites.

### Predator models

We used models of naturally occurring predators to examine the escape trajectories of túngara frogs in response to aerial and terrestrial predators. A major aerial predator of túngara frogs is the fringed-lipped bat, which is attracted to the frogs’ advertisement calls [31]. We created a model of this bat from a traced outline of a preserved specimen. Similar bat models have been successfully used to elicit anti-predatory behaviours in túngara frogs [32]. We deployed the model using a 2.95 m zip line at an approximately 50° angle, which passed directly over the calling male frog. To ensure that the experimenter was not within visual range of the frog, the bat model was deployed using a remote release system. The end points of the zip line were fixed in height but depending on the position of the frog between the start and endpoints, the height of the model above the frog varied from 25–86 cm with a median height of 40 cm. We directly measured height immediately after each trial and not before, so as not to disturb the calling behaviour of the test frog. A total of 24 frogs were exposed to the bat model; 19 responded to the model by escaping. To examine consistency in the angle of escape in response, we presented the bat model a second time after the frog had resumed calling. Thirteen individuals, out of 19, fled during a second trial.

We simulated a ground predator using a rubber snake equivalent in size and shape to a cat-eye snake *Leptodeira annulata* (45.5–61 cm), another known predator of calling túngara frogs [24]. We placed the snake model at 1–1.5 m from the calling male and dragged it towards the frog at a speed of 22±8 cm/sec using a monofilament. The frog was unable to see the filament as evidenced by a lack of response when the filament was waved directly in front of the frog. The snake model was dragged such that the experimenter was not visible to the frogs.

Both model predators were only presented to actively calling male frogs. Given that frogs attacked by bat models remained in the immediate vicinity and would often resume calling after a silent interval, we tested each frog with the bat model twice. When presented with the snake model, however, frogs moved farther away than when presented with the bat model. The frogs exposed to the snake model were often difficult to locate once they fled and did not resume calling. For this reason, frogs presented with the snake model could only be tested once.

### Statistical analyses

We transformed digitised x-y coordinates from the video into polar coordinates using an arctan transformation. We calculated the extent to which the frog rotated in response to the model as the difference between the directions the frog faced before and after exposure to the stimulus. Frog flight paths were expressed relative to the model’s approach trajectory. A 0° escape corresponds to the frog moving directly toward the model while 180° represents the frog fleeing directly away from it. A negative or positive prefix refers to the frog fleeing to the left or right of the approaching axis of the predator.

To determine if the angle the frog faced influenced the direction the frog fled we conducted a correlation test for circular data following [33]. To examine if the height at which the bat model flew over the frog influenced the angle of escape we used a Johnson-Wehrly-Mardia Correlation test using method outlined [34]. We used a one-way binomial test to test the a priori prediction that the frogs would move away from the model, a prediction based on many previous studies that have examined animal escape responses [reviewed in 12]. We classified individuals as moving away or towards the model if their flight trajectories were 90–180° or 0–90° respectively. We statistically tested if the flight path of the frogs is directional (i.e. paths constricted between a small range of angles) or chosen at random (i.e. paths distributed as a wagon wheel [10]) by testing for circular uniformity of the escape directions using Watson tests conducted on unbinned data. We also used Watson tests to examine normality; we conducted these tests on pooled data comparing the realised flight distribution with a von Mises distribution (i.e. circular normal distribution) [35]. Following [16], we pooled data within 0–180° whereby left and right escape directions were treated as if the frog always fled to the right of the predator from the predator’s point of view. This approach ultimately assumes the angle of jump relative to the model trajectories is not influenced by a left or right bias.

To compare the circular distributions between predator models, we employed the Watson’s two-sample test of homogeneity, which is appropriate for non-normal data and, is robust for small sample sizes [33]. Frog escape trajectories in response to the bat model were bimodally distributed, with four of 19 trajectories away from the model. Following the suggestion by [33] and the subsequent applied approach by [36], separate hemispheres were treated independently. Given the presence of rare away responses, we qualitatively assessed the distribution of escape angles representing them graphically. We tested the repeatability of the escape response of the frog to the bat model using a Moore’s test for paired circular data as outlined by [34]. All descriptive and test statistics on circular data were calculated using the R package ‘Circular’ v 0.4–1 [37]. We analysed differences in time to flight using a Mann-Whitney U-test as the data violated the assumptions of normality. All summary statistics are reported as means and standard errors except for escape angles for which medians and upper and lower quartiles are reported to account for non-normality.

All animal handling and experimental procedures were reviewed and approved by the Smithsonian Tropical Research Institute IACUC #2011-0616-2014-11. Permits were granted by the Autoridad Nacional del Ambiente (ANAM, #SE/A-57-11).

## Results

Individuals threatened by the approaching predator model would sequentially perform the following defensive behaviours: (1) cease calling keeping their body still inflated (deflated vocal sac), (2) cease calling and rapidly deflate both their body and vocal sac, (3) jump to safety or dive under the water surface. This observed graded defensive response is consistent with those reported in previous experiments using predator models with túngara frogs [32]. The frogs did not flee when the bat model passed above the frog at a height exceeding 50 cm instead the frog ceased calling with or without deflating its body (call cessation only: N_ind_ = 10, n_trials_ = 17, 59.3±3.6 cm; call cessation and fleeing: N_ind_ = 21, n_trials_ = 38, 39.5±1.8 cm). The height at which the bat model flew above the frog did not, however, influence the escape angle of fleeing individuals (N = 19, R^2^ = 0.21, P(9999) = 0.15). In response to the snake model, all individuals responded by fleeing. The time from predator detection to fleeing, however, was significantly longer in frogs exposed to the snake model than in response to the bat model (N_bats_ = 19, 0.24±0.06 sec; N_snake_ = 17, 3.68±0.89 sec; Mann-Whitney test: U = 20.5, df = 1, p<0.001).

**Fig 1:**
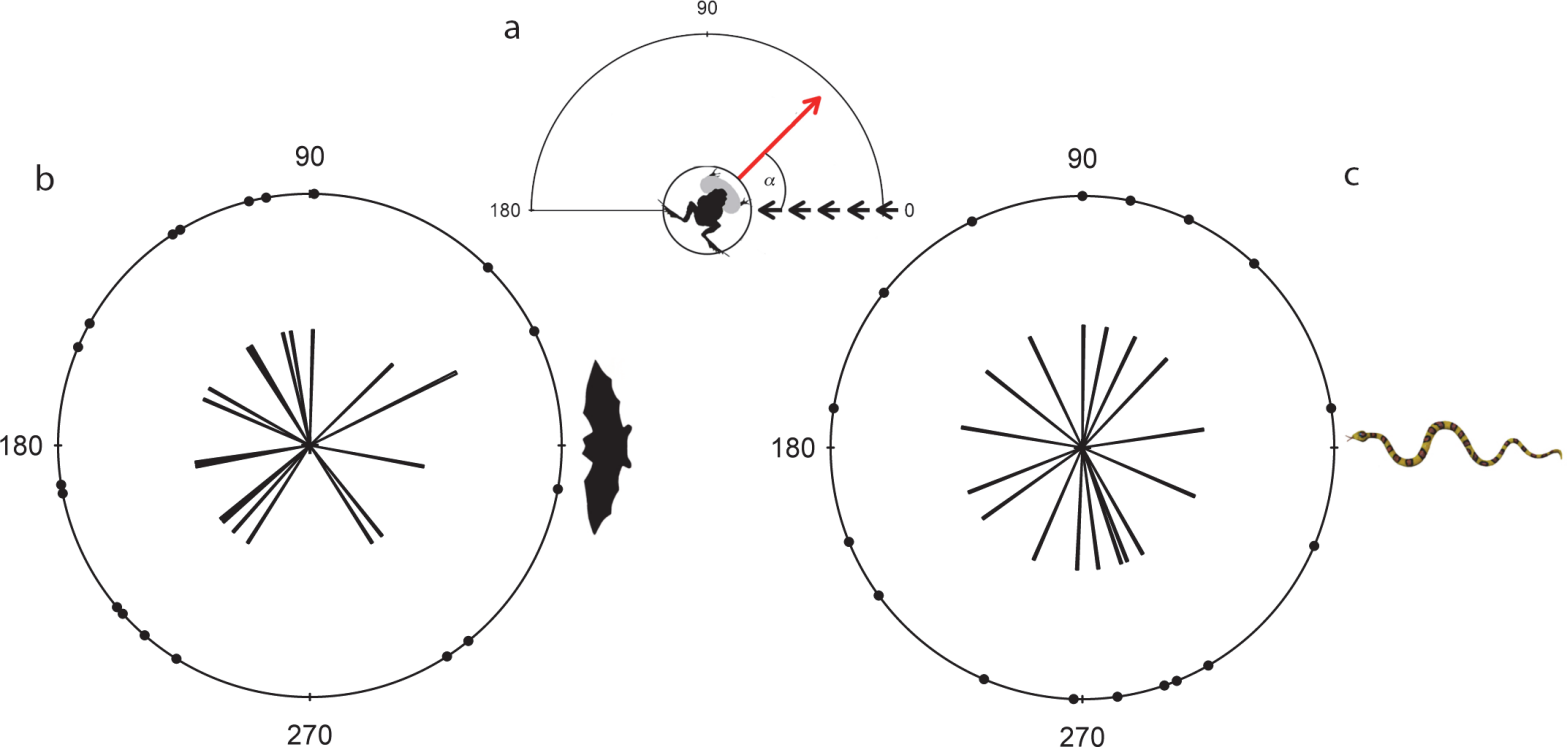
Circular distribution of the directions each frog faced immediately prior to onset of trial. (a) Schematic illustrates the method used to measure the initial frog orientation relative to the predator model’s direction of approach; Short black arrows indicate trajectory of predator approach at 0°; Long arrow indicates prey orientation while angle α indicates the prey’s angle of deviation from the predator approach. Orientation angle distribution of frogs presented with bat (b) and snake (c) models was random for both predators.

**Fig 2:**
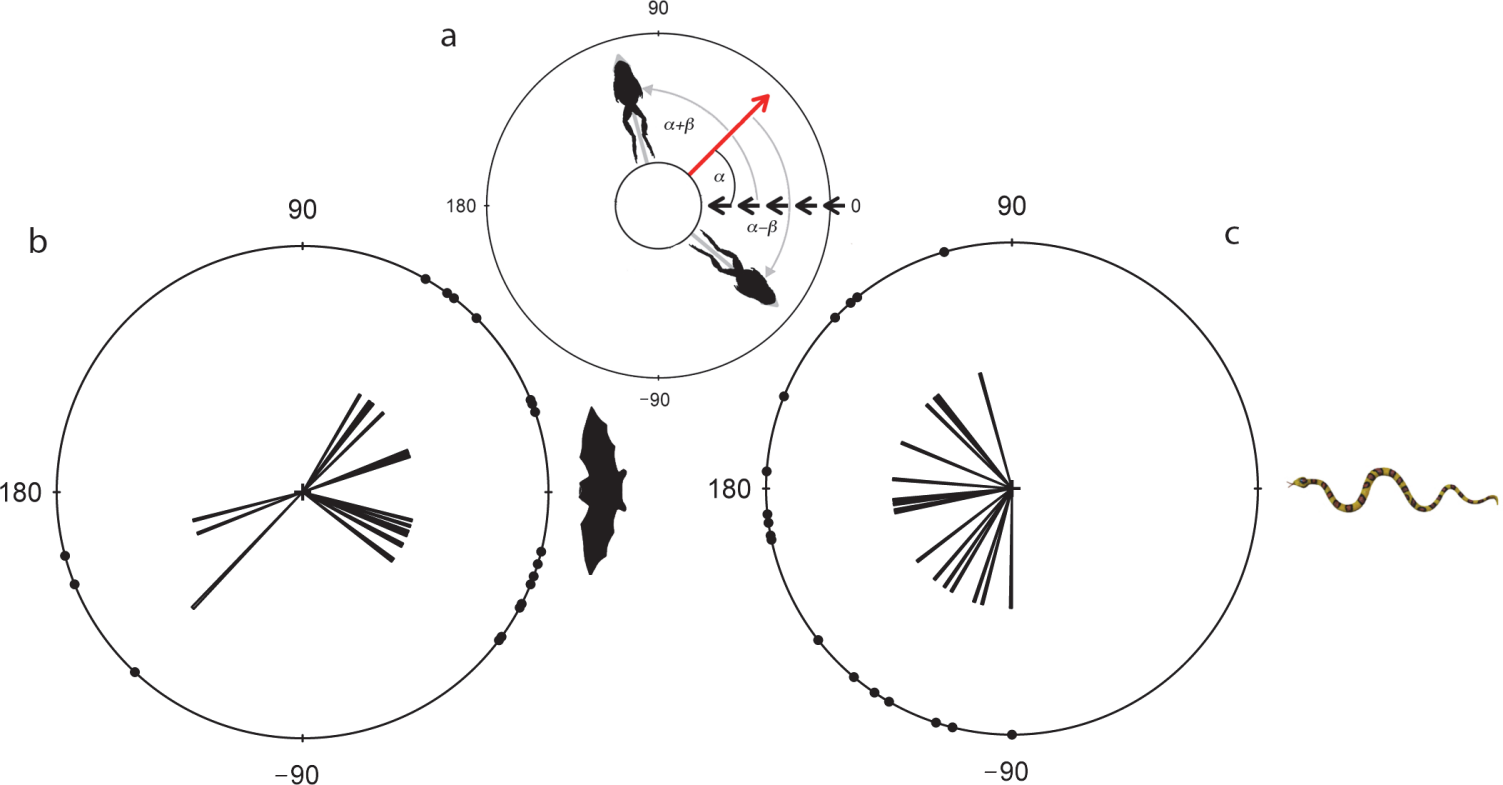
Escape trajectories of túngara frogs relative to the predator approach. (a) Schematic illustrates method of measuring the angle for fleeing frogs relative to the predator model; Short black arrows indicate trajectory of predator approach at 0°; Long arrow indicates prey orientation while angle α indicates the prey’s angle of deviation from the predator approach. Angles α+β and α-β are the resultant angles of flight relative to their initial orientation of the prey to the bat (b) and snake (c) models.

The angles at which the model approached the frogs were random and homogenous, with no directionality for either the bat (n = 19, *U* ^2^ = 0.0591, P > 0.1; Fig. 1B) or snake models (n = 17, *U* ^2^ = 0.027, P > 0.1; Fig. 1C). There was also no significant difference in the distribution of approach angles between the trials using bat versus snake models (Watson two-test: *U* ^2^ = 0.065, P > 0.1). In contrast, the escape response angles by frogs to both the bat and snake models were directional (bat: n = 19, *U* ^2^ = 0.315, P < 0.01; snake: n = 17, *U* ^2^ = 0.434, P < 0.01; Fig. 2) and significantly different to each other (Watson two-test: *U* ^2^ = 0.396, P < 0.001).

**Fig 3:**
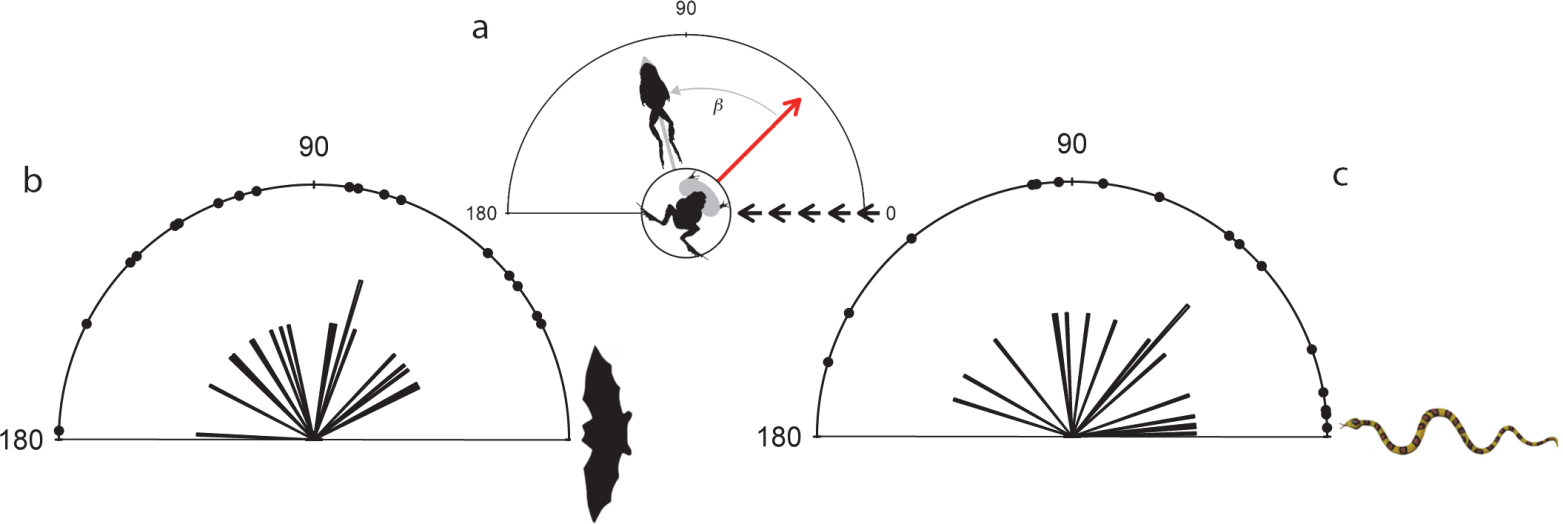
The extent the túngara frogs rotated to reach their resultant escape angles. (a) Schematic illustrates the method for calculating how far the frog rotated when fleeing; β indicates the angle moved by the frog relative to its initial position. There was no significant difference in fleeing angle between frogs presented with bat (b) and snake (c) models.

The direction the frog faced when threatened did not dictate the direction in which it escaped (bat: Cor = 0.11, Stat = 0.51, P = 0.61; snake: Cor = 0.082, Stat = 0.37, P = 0.737). The frogs actively rotated their bodies until reaching a direction to escape. The angles of rotation for both the bat and snake models did not vary from random (test for normality: bat: n = 19, *U* ^2^ = 0.048, P > 0.1; snake: n = 17, *U* ^2^ = 0.049, P > 0.1). Additionally, the angles of frog rotation for both predator models were not significantly different to each other (Watson two test: *U* ^2^ = 0.075, P < 0.01; Fig. 3). When taking into account the angle of approach of the predator, however, there was a difference in the degree of rotation of the frog relative to the bat or snake model. The greater the starting angle of the frog relative to the bat model, the greater the angle of rotation before flight (Cor = 0.66, Stat = 2.77, P = 0.006). In contrast, when the frogs were threatened with the snake model, the greater the angle of the frog relative to the snake the lower the angle of rotation (Cor = -0.84, Stat = -2.82, P = 0.005).

**Fig 4:**
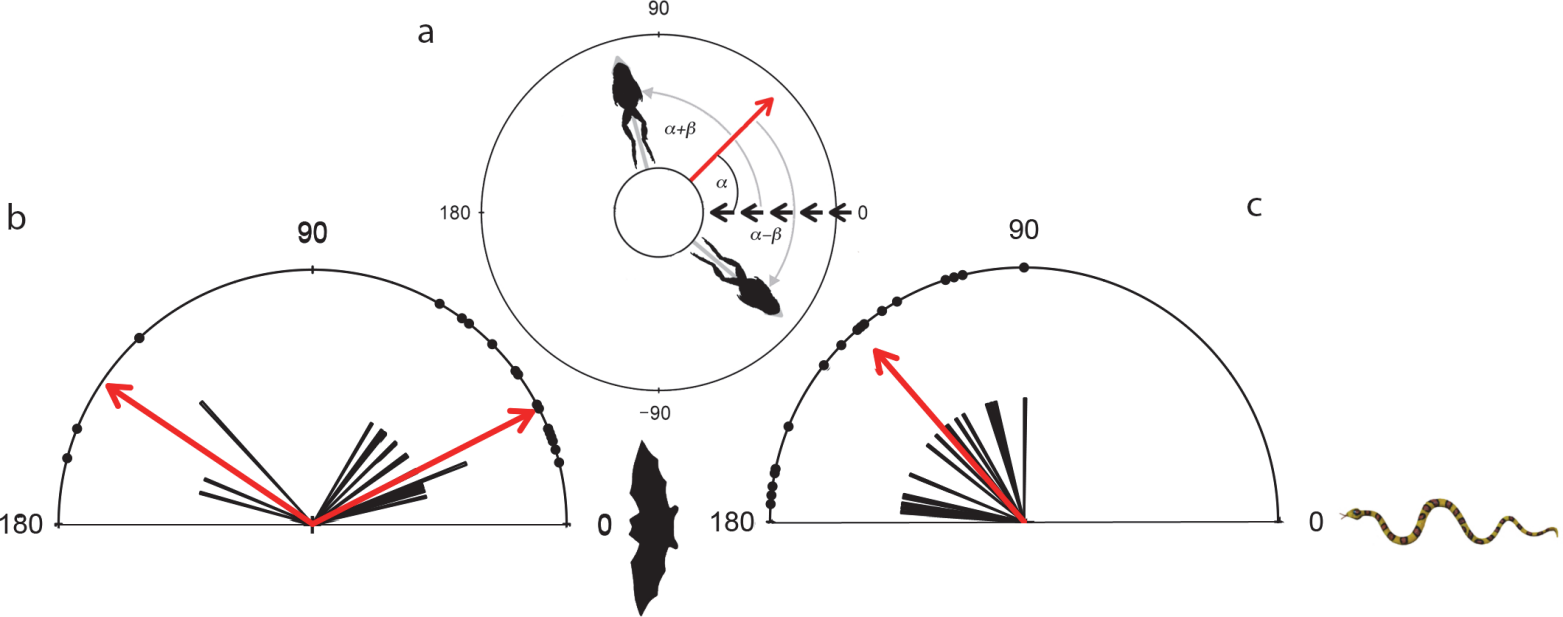
Randomness of escape trajectories of túngara frogs. (a) Schematic illustrates method of measuring the angle for fleeing frogs relative to the predator model; Short black arrows indicate trajectory of predator approach at 0°; Long arrow indicates prey orientation while angle α indicates the prey’s angle of deviation from the predator approach. Angles α+β and α-β are the resultant angles of flight relative to their initial orientation of the prey to the predator. Escapes to the left and right of the predator were pooled as if the escapes were always to the predator's right. (b) and (c) are pooled data for the bat and snake respectively with the arrow indicating the median resultant angle of flight.

Unexpectedly, the frogs exposed to the bat model did not move away from the model (one-way binomial test: 4/19, P = 0.998, probability of fleeing away from the bat = 21%). Instead, the frogs fled at angles ranging between 10–60° relative to the bat model’s angle of approach (n = 15, rho = 0.969, Median angle = 27°, Quartiles: Upper = 60°, Lower = 14°; Fig. 4B). There was a noticeable absence of flight angles ±10^o^ to the models pathway suggesting that the frogs did not move directly at the model but slightly to either side of the approaching bat (Fig. 2B). For the frogs subjected to the bat model twice, the escape angles of repeated trials were slightly more acute (n = 13, rho = 0.962, Median angle = 15°, Quartiles: Upper = 64°, Lower = 6°) but the distribution did not differ significantly from the distribution in the first set of trials (Moore test: n = 13, Test statistic = 0.765 P = 0.203). In four of the 19 trials, however, frogs responded by escaping away from the bat model (n = 4, rho = 0.968, Median angle = 146°, Quartiles: Upper = 165°, Lower = 133°; Fig. 4B). In all of these cases, there was an obvious refuge nearby under which the animals dived. In contrast, no refuges were apparent during trials in which the frogs jumped towards the bat.

The snake model induced more conventional escape responses. As predicted, frogs moved away from the snake model (binomial test: 16/17, P = <<0.001, probability of fleeing away from the snake = 94%; Fig. 4C) with escape angles between 90–180° (n = 17, rho = 0.962, Median angle = 131°, Quartiles: Upper = 176°, Lower = 90°) relative to the model.

## Discussion

Our results show predator-specific differences in escape directions of prey in response to predators with alternative attack trajectories. As predicted, túngara frogs fled away from the approaching snake model (Median: 131°), which concurs with findings from previous studies that have used snake models [38] or observed responses to real snakes [39,40]. In contrast, and contrary to expectation, the frogs moved toward the bat model at an acute angle (Median: 27°) relative to the angle of predator approach. Previous studies have examined the responses of a single prey species to either aerial or terrestrial predators, suggesting differential strategies to these two predator types (e.g. [14,21,41–43]). In these studies, however, differences in prey escape response could be due to prey-specific behaviour. In our study, by examining the escape response of a single prey species to both aerial and terrestrial predators, we demonstrate that prey indeed show predator-specific escape trajectories.

Studies to date have shown that prey escape trajectories are characteristically constrained to the hemisphere opposite to the threat (90–180°); that is, fleeing in a direction opposite or perpendicular to the predator’s approach (reviewed in [12]). Escape trajectories outside this range of angles are seldom observed and often attributed to a prey’s error in judgement [44,45]. A notable exception are juvenile Algerian lizards (*Psammodromus algirus*), which are seemingly ‘hard-wired’ to escape in the direction they are facing when attacked even though this strategy at times puts them in the path of the predator ([46]. Similarly, gerbils (*Meriones unguiculatus*) respond to a non-ecologically relevant stimulus that approaches from above by fleeing in the direction they are facing resulting in some escape trajectories in the direction of the approaching object [47]. Túngara frogs, however, escaped from the bat model by moving toward the direction of the threat. This behaviour was not a limitation of their initial orientation as their escape trajectory was independent of the direction the frog was facing prior to the attack. It is also unlikely that this behaviour was due to an error of judgement given that the frogs adjusted their body orientation to consistently reach escape angles towards the bat model. When initially facing away from the model, túngara frogs rapidly rotate their bodies up to ~180° to face the predator and move toward it. Although active selection of an escape angle by rotating the body takes valuable time away from escaping, it is probably a more widespread phenomenon than previously anticipated.

Flexibility in prey fleeing response, such as the behaviour described in this study, is widespread across prey species. For instance, male great tits (*Parus major*) presented with a raptor model at a range of different aerial angles of approach modulate their take-off angle. Great tits take off at shallower angles in response to predators with steep attack trajectories (45°), while they use higher angles in response to the same predator approaching with shallow attack trajectories (15°) [48]. Our results are similar in showing flexibility in prey response; however, rather than subtle changes over a few degrees in escape response, we show a dichotomy in response with túngara frogs fleeing away from terrestrial predators and toward aerial ones.

While movement toward an approaching predator is risky, it is possible that it is the most effective method of predator avoidance when prey cannot outpace their attacker. It is common that prey adjust the flight initiation distances to outpace the predators they confront [4,49]. Fleeing prey species, however, are often unable to match the high speeds of aerial predators in pursuit [21,50–52]. Consequently, such prey rely on out-manoeuvring the predator rather than initially outpacing them [42,52]. It is unlikely that a túngara frog could outpace an attacking bat. Instead, by moving towards the approaching bat, túngara frogs may out-manoeuvre their aerial predator. Consistently, dead mice dragged towards swooping barn owls (*Tyto alba*) avoid capture more often than mice dragged away from them [15]. Although those results are in agreement with the interpretation that approaching the predator increases survival rate, prey of barn owls escape by moving perpendicular rather than towards the approaching predator [14]. Further studies that examine the manoeuvring ability of predators and prey are necessary to better understand the strategies shaping prey escape directions.

In túngara frogs, several lines of evidence suggest that preferentially moving towards an aerial threat likely increases the rate of successful escape. First, the resultant fleeing velocity of the frog escaping towards the predator is higher than the resultant velocity the frog could achieve by moving away from it given the combined velocity of the approaching predator and the prey moving in the same direction. Second, the frog effectively increases its distance from the bat without having to cover as much ground (i.e. combined distance the frog jumps and the distance the bat overshoots the capture site before turning). Together, increased relative fleeing velocity and increased fleeing distance provide the bat with less time to access and respond to the prey than if the prey moves away or perpendicular to the predator. Once the frog moves toward the bat and the bat passes over the frog, the frog stops moving and remains silent. Fringe-lipped bats rely heavily on prey-emitted acoustic cues to localise their prey [53], and like many gleaning bat species, are limited in their ability to locate prey by echolocation alone in the clutter of the dense forest understory [54–56]. By undercutting the bat’s approach path the frog effectively moves to a ‘temporal refuge’ by remaining silent and motionless, the frog increases its chance of escape.

It is well documented that frogs typically flee away from attacking terrestrial predators [38,39,57,58], which our study further supports. In contrast, the influence of aerial attacks on frog escape strategies has received little attention even though anurans are often susceptible to attacks by both birds [59,60] and bats [23]. The few studies investigating the response to aerial threats suggest that escape trajectories in response to aerial predators are more variable than escape trajectories in response to terrestrial ones. Several species of cryptic robber frogs (*Craugastor* spp), for instance, presented with metallic objects moving towards them from various elevations, jumped away or had random escape trajectories relative to the angle at which the object approached [9]. In contrast, Northern leopard frogs (*Rana pipens*), tested in response to black squares moved in a pendulum fashion towards the frog, consistently responded by turning away from the object [61,62]. How results from these collision avoidance experiments can be interpreted in light of aerial predator escape behaviour is unclear, however. Studies using ecologically relevant stimuli may shed more light on escape strategies and reveal unexpected responses such as the ones found in túngara frogs.

In this study, túngara frogs occupied human-constructed drainage lines. Although these calling sites are typical for this species often found in urban environments, it is unclear whether túngara frogs show similar escape responses in more natural settings. As many other animals, frogs have ‘instinctual’ spatial awareness to avoid known physical barriers when escaping [61]. Unexpectedly acute escape angles towards the bat model could result from túngara frogs avoiding a collision with a wall. Even if the walls are responsible for polarising the responses to the predator models more strongly, different responses to the aerial and terrestrial predator models are unlikely to result from calling in the drainage lines. In addition, evidence from object collision studies suggests our findings represent a widespread phenomenon. For example, American bullfrogs (*Lithobates catesbeianus*) respond differently to a simulated object moving towards the frog from different areas in their visual field. When the stimulus was presented in the frontal visual field, the frog jumped forward. In contrast, the individuals turned and jumped towards the stimulus when it was presented in the caudal visual field [63]. These responses are equivalent to the escape trajectories of túngara frogs observed in our study.

To our knowledge, ours is the first study to investigate the escape trajectories of a single prey species confronted with both terrestrial and aerial simulated predators in the prey’s home environment. Our study demonstrates that túngara frogs use different escape trajectories in response to attacks by bats and snakes. In nature, prey confront multiple predators that use different attack tactics [64]. Predator-specific responses like the ones found here are potentially widespread among prey and have been missed due to the subtlety of altering a flight path. This study thus emphasizes that flexibility in prey escape trajectories is a potentially simple but highly effective strategy that could allow prey to effectively outcompete multiple predator types.
